# Factors influencing the use of highly bioavailable oral antibiotic therapy for the treatment of prosthetic joint infections

**DOI:** 10.1017/ice.2024.159

**Published:** 2024-11

**Authors:** Marie-Félixe Granger, Jerome A. Leis, Amanda Hempel, Daniel Pincus, Bheeshma Ravi, Nick Daneman, Philip W. Lam

**Affiliations:** 1 Temerty Faculty of Medicine, University of Toronto, Toronto, ON, Canada; 2 Division of Infectious Diseases, Sunnybrook Health Sciences Centre, Toronto, ON, Canada; 3 Institute of Health Policy, Management and Evaluation, and Centre for Quality Improvement and Patient Safety, University of Toronto, Toronto, ON, Canada; 4 Division of Orthopaedic Surgery, University of Toronto, Toronto, ON, Canada

## Abstract

We conducted a retrospective cohort study to identify factors influencing intravenous (IV) versus oral antibiotic therapy in first-episode prosthetic joint infections. Of the 34/78 (44%) cases treated intravenously, negative cultures (26%), concomitant infections necessitating IV antibiotics (21%), and delays in susceptibility testing (15%) were the most common reasons for IV therapy.

## Introduction

Bone and joint infections, including prosthetic joint infections (PJIs), have historically been managed with intravenous (IV) antimicrobial therapy. More recently, evidence suggests that antibiotics with excellent oral bioavailability are as effective as IV in the treatment of osteoarticular infections.^1–4^ Oral therapy carries potential benefits including avoidance of vascular catheters and their associated complications, lower resource utilization, and higher patient quality of life.^5–7^

Translating this knowledge into practice has not been universal with many clinicians still routinely using IV antibiotics for PJI.^8^ A limited number of real-world observational studies describe early transition from IV to oral therapy,^1,2,6,7^ but few of them assess factors influencing this transition. Such studies can help identify potential barriers to using oral antibiotics in PJI and improve knowledge translation.

We previously implemented a quality improvement intervention to promote use of oral therapy for PJI at our institution. The following retrospective cohort study was conducted to examine factors that influence the choice of IV versus oral therapy.

## Methods

Sunnybrook Health Sciences Centre (SHSC) is an academic health sciences center located in Toronto, Canada where over 6000 orthopedic surgeries are performed annually. Evidence supporting the use of highly bioavailable oral antibiotic therapy for PJI was presented to infectious diseases (ID) and orthopedic surgery stakeholders in November 2021. A standardized process for the perioperative management of PJI was developed and implemented in February 2022. This process incorporated practice changes that supported the appropriate use of oral antibiotic therapy. All PJI cases were assessed by ID physicians who provided recommendations on empiric and definitive treatment, and appropriateness for transition to highly bioavailable oral therapy. This initiative was focused on improving oral antibiotic therapy for first-episode PJI, in order to help clinicians gain comfort in more straightforward cases of infection.

A retrospective cohort study examining patients with first-episode of PJI at SHSC was conducted from February 1^st^, 2022 to January 31^st^, 2024. All revision arthroplasty cases during the study period were reviewed to determine the indication for revision. Only revision cases for first-episode PJI were included. For each PJI, the following variables were collected: age, sex, date of the surgery, anatomical site of the prosthesis, revision type (1 stage, 1.5 stage, 2 stage, or debridement, antibiotics, and implant retention), orthopedic surgeon and ID consultant involved, and definitive antibiotic treatment. Based on the duration of IV treatment in the oral versus intravenous antibiotics (OVIVA) for bone and joint infection trial,^3^ definitive treatment was defined as the antibiotic prescribed on the 7^th^ postoperative day. We established this timepoint to identify cases where delays in obtaining susceptibilities were the cause of postponing oral therapy. For all PJI treated with definitive IV therapy, the reason was determined through chart review and grouped into major themes. When it was unclear, the ID consultant involved in the case was interviewed.

The primary outcome was the reason why patients with first-episode of PJI received definitive IV instead of oral antibiotic therapy. Descriptive statistics were used to characterize the cohort. Medians and proportions were calculated for continuous and categorical variables, respectively. Research ethics review was exempted based on our institutional process which confirmed that the project was deemed improvement in quality and not human subject research.

## Results

There were 108 revision arthroplasties for PJI performed during the study period, of which 78(72%) were performed for a first-episode PJI and included in the analysis. Table 1 outlines the clinical characteristics of the cohort, stratified by the route of definitive antibiotic. The median age was 72 years with more women than men. Approximately the same number of hips and knees were operated [39(50%) and 37(47%), respectively]. In total, 44(56%) and 34(44%) patients received definitive oral and IV therapy, respectively.

**Table 1.** Baseline characteristics of patients who received intravenous versus highly bioavailable oral antibiotic therapy for prosthetic joint infection

	Entire Cohort (n = 78)	Definitive Oral Antibiotics (n = 44)	Definitive IV Antibiotics (n = 34)
**Age** (median)	72	72	70
**Sex**			
Female	43	23	20
Male	35	21	14
**Type of joint infected**			
Hip	39	23	16
Knee	37	19	18
Shoulder	2	2	0
**Type of revision**			
1 Stage	29	18	11
1.5 Stage^#^	3	2	1
2 Stage	7	5	2
DAIR	39	19	20
**Pathogens**			
Coagulase negative *Staphylococcus spp*	24	17	7
*Staphylococcus aureus* methicillin-susceptible (MSSA)	14	6	8
*Staphylococcus aureus* methicillin-resistant (MRSA)	4	2	2
β-hemolytic *Streptococcus spp*	5	3	2
Viridans *Streptococcus spp*	4	1	3
*Enterococcus spp*	3	2	1
*Cutibacterium spp*	10	7	3
*Enterobacterales*	5	5	0
*Pseudomonas aeruginosa*	3	1	2
Other^*^	7	2	5
Culture negative	8	2	6
Mixed growth	7	3	4
**Antibiotic Class**			
Fluoroquinolone	17	17	0
Penicillins	7	5	2
Cephalosporins	20	4	16
Carbapenem	3		3
Co-trimoxazole	0	0	0
Doxycycline	24	24	0
Rifampicin (in combination)	6	4	2
Glycopeptide	16		16
Combination therapy	16	10	6

# Defined as placement of a functional articulating antibiotic spacer intended to remain in place indefinitely.

* Other bacteria isolated include: Pseudomonas fluorescens, Listeria monocytogenes, Ralstonia spp., Corynebacterium spp.

A similar number of patients with retained prosthetic material were treated with oral [19/39(49%)] and IV [20/39(51%)] therapy. The most common pathogens isolated were coagulase-negative *Staphylococcus spp* (31%)*, Cutibacterium spp* (13%), and *Staphylococcus aureus* (23%). While coagulase-negative *Staphylococcus spp*, *Cutibacterium spp,* and *Enterobacterales* were mostly treated orally, only half of the *S. aureus* cases were treated with oral therapy. Culture-negative infections were mainly treated intravenously [6/8(75%)]. The most commonly prescribed oral antibiotics were fluoroquinolone [17(39%)] and doxycycline [24(55%)]. Of the 10 patients who received combination oral therapy, 8 were treated with doxycycline and a fluoroquinolone. The remaining two received a combination of rifampicin with either fluoroquinolone or doxycycline.

Of the patients who received IV antibiotics, the rationale for prescribing IV over oral therapy was identified in all cases (Table 2). The three most common reasons were a negative culture [9(26%)], a concomitant infection necessitating IV antibiotics [7(21%)], and a delay in obtaining susceptibility testing for oral agents [5(15%)]. A surgeon’s preference for IV treatment occurred 4 times (12%) but no orthopedic surgeon or ID consultant consistently preferred IV over oral antibiotic therapy.

**Table 2.** Reasons for using intravenous (IV) instead of oral antibiotics for patients with first-episode prosthetic joint infection

Reason	Frequency n (%)
Negative culture	9(26.5)
Concomitant infection necessitating IV antibiotic^†^	7(20.5)
Delay in obtaining susceptibility results	5(14.7)
Intolerance	4(11.7)
Surgeon’s preference	4(11.7)
Bacteria where IV treatment preferred due to resistance, or limited evidence supporting oral therapy ^#^	3(8.8)
Infectious diseases specialist’s preference	2(5.9)

† Concomitant infections included: bacteremia due to Staphylococcus aureus (4), Streptococcus gallolyticus (1), Pseudomonas aeruginosa (1), and intra-abdominal abscess without adequate source control (1).

# Bacteria in this category included: Listeria monocytogenes, Ralstonia spp, and methicillin-resistant Staphylococcus species.

## Discussion

Following the implementation of local practice change for the management of first-episode PJIs, about half of the patients were successfully transitioned to definitive therapy using highly bioavailable oral antibiotics. This real-world experience following previous randomized controlled trials^3,4,9^ provides a minimum achievable target and highlights some opportunities to further increase the use of these agents in PJI management.

Few studies have addressed the reasons that lead to the selection of IV over highly bioavailable oral therapy in spite of non-inferiority and increased patient risks associated with the former. Halouska et al found similar rates of oral antibiotic therapy for PJI and suggested that providers’ apprehension was the main factor driving the continuation of IV therapy.^2^ Azamgarhi et al found that among the one-third of patients who remained on IV antibiotics in their study, the two most common reasons were antibiotic resistance and culture-negative infection.^1^ Similarly, our study identified negative cultures as one of the top reasons for choosing definitive IV therapy.

Our study identifies potential barriers to improve knowledge translation of previous randomized controlled trials. Some barriers are unavoidable such as concomitant infection necessitating IV antibiotics, allergy, and resistance to highly bioavailable oral options. Yet even after considering these causes, we found that around 60% of the cases represented avoidable barriers, and therefore opportunities to improve the use of highly bioavailable agents. In contrast to what might be assumed for those undergoing practice change, we did not identify concerns from specific clinicians about using oral antibiotics.

Culture-negative infections represent a significant opportunity to develop empiric combination oral therapy treatment regimens through the use of weighted-incidence syndromic combination antibiograms.^10^ Reducing the delay in obtaining extended antibiotic susceptibility testing results is another opportunity to facilitate practice change in partnership with the microbiology laboratory. One strategy could include reporting validated oral antibiotics in the first-line antimicrobial panel for bone and joint specimens.

Our study has limitations. First, the small sample size in this single-center study limited our ability to perform additional analyses. Secondly, the degree of oral antibiotic use may differ in centers where ID consultations are not routinely sought for all PJI cases. Finally, our study only included first-episode PJIs. As a result, additional factors influencing antibiotic choice in recurrent PJIs were not explored.

Given the benefits of using highly bioavailable therapy over IV treatment of PJI, understanding barriers to practice change is vital to knowledge translation. Our study identifies key considerations for institutions hoping to optimize the uptake of the highly bioavailable oral antibiotic therapy for PJI.
